# Supplementary material for: A Compositional Heterogeneity Analysis of Mitochondrial Phylogenomics in Chalcidoidea Involving Two Newly Sequenced Mitogenomes of Eupelminae (Hymenoptera: Chalcidoidea)
Source: Genes (Basel). 2022 Dec 11;13(12):2340. doi: 10.3390/genes13122340 (PMC9778353; doi:10.3390/genes13122340)
Supplement: Supplementary file 1 [file genes-13-02340-s001.zip › genes-1967462-supplementary.pdf]

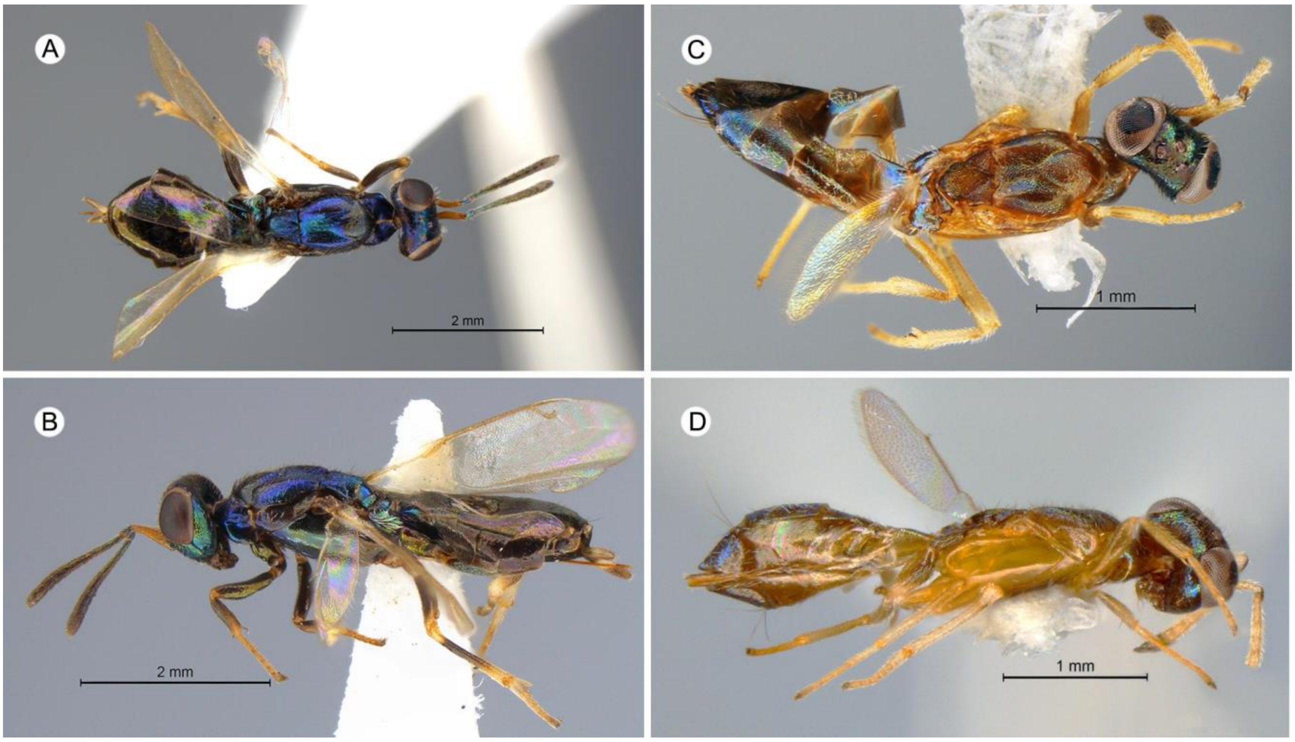

Figure S1. Dorsal and lateral views of *Eupelmus anpingensis* and *Merostenus* sp. *Eupelmus anpingensis* (A & B); *Merostenus* sp. (C & D)

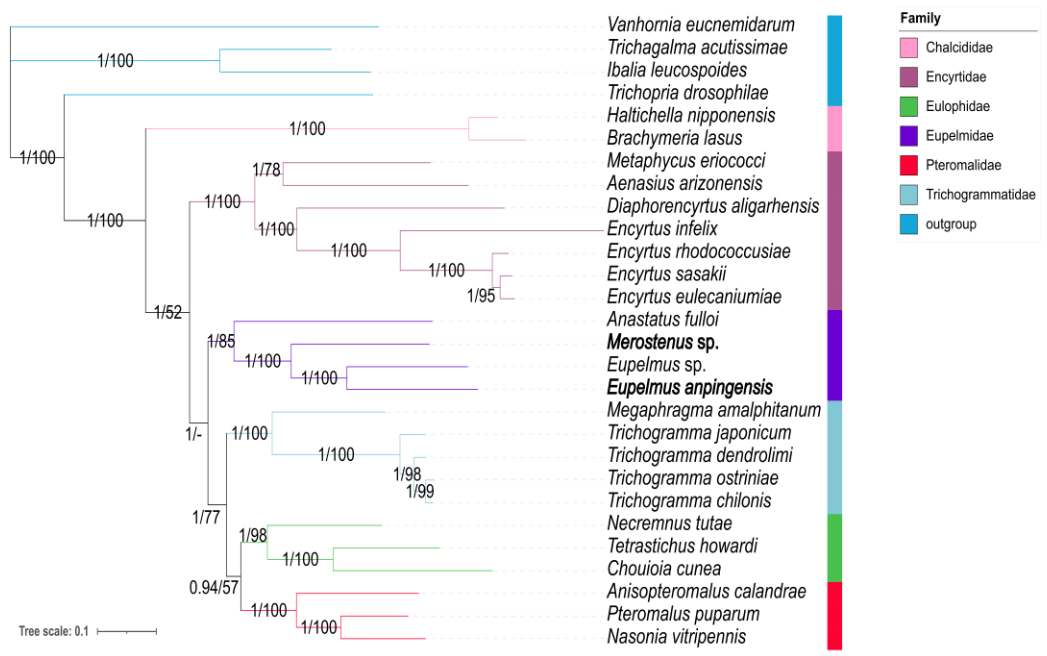

Figure S2. Phylogenetic tree inferred from MrBayes and IQ-tree based on the datasets of AA. Supports at nodes (from left to right) are Bayesian posterior probabilities (PP) and ML bootstrap support values (BS).

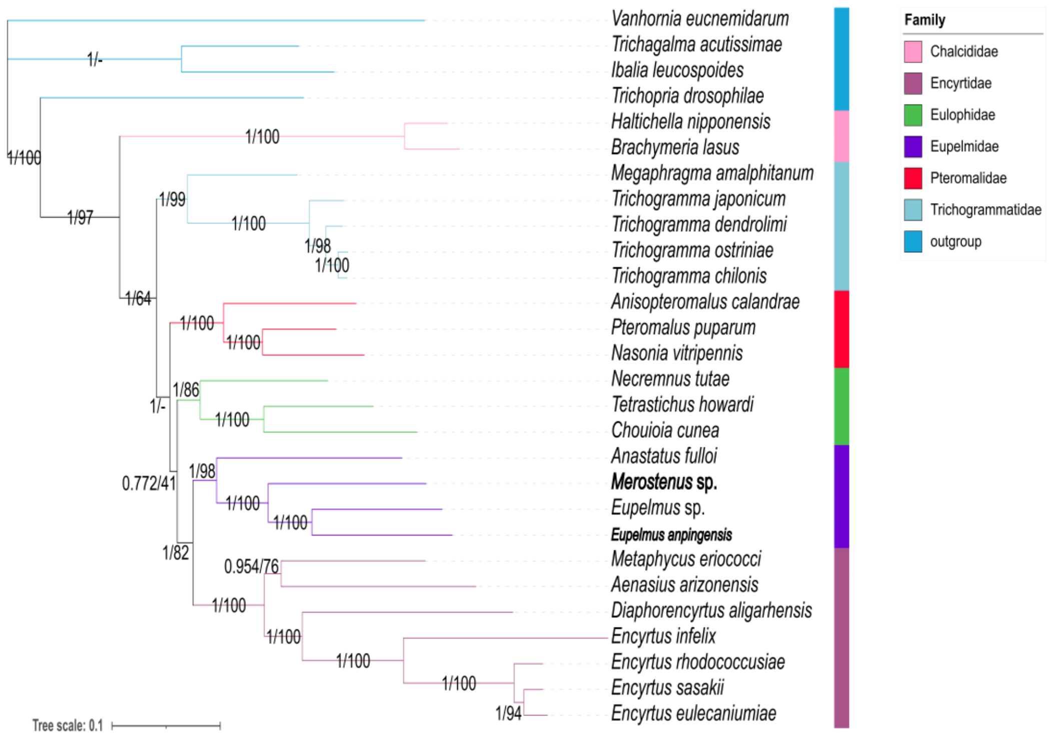

Figure S3. Phylogenetic tree inferred from MrBayes and IQ-tree based on the datasets of PCG12. Supports at nodes (from left to right) are Bayesian posterior probabilities (PP) and ML bootstrap support values (BS).

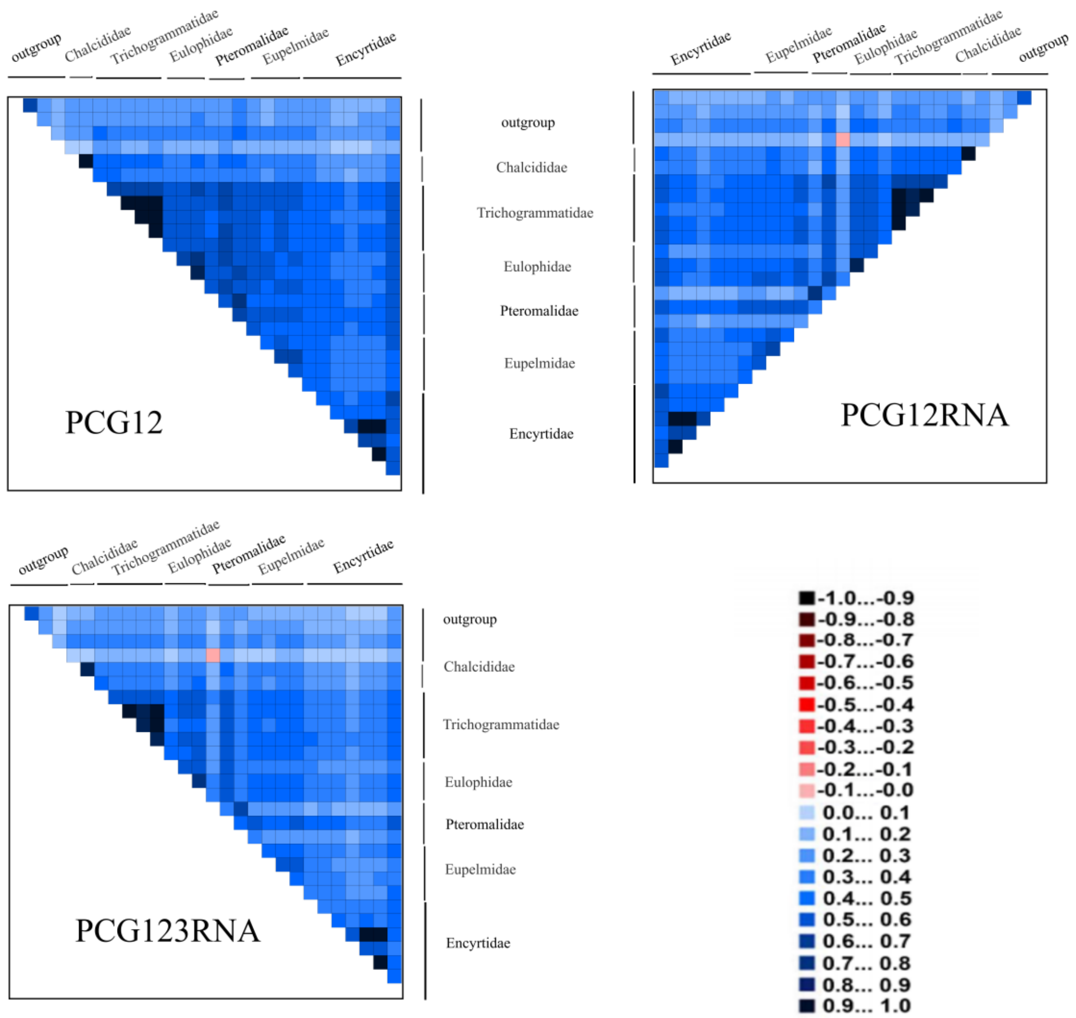

Figure S4. Heterogeneous analysis of 28species (including four outgroups) based on PCG12, PCG12RNA and PCG123RNA datasets. The mean similarity score between sequences is represented by a colored square. AliGROOVE score rang from -1, which indicates distances are very different from the average for the entire data matrix (red color), to +1, which indicates distances match the average for the entire matrix (blue color)

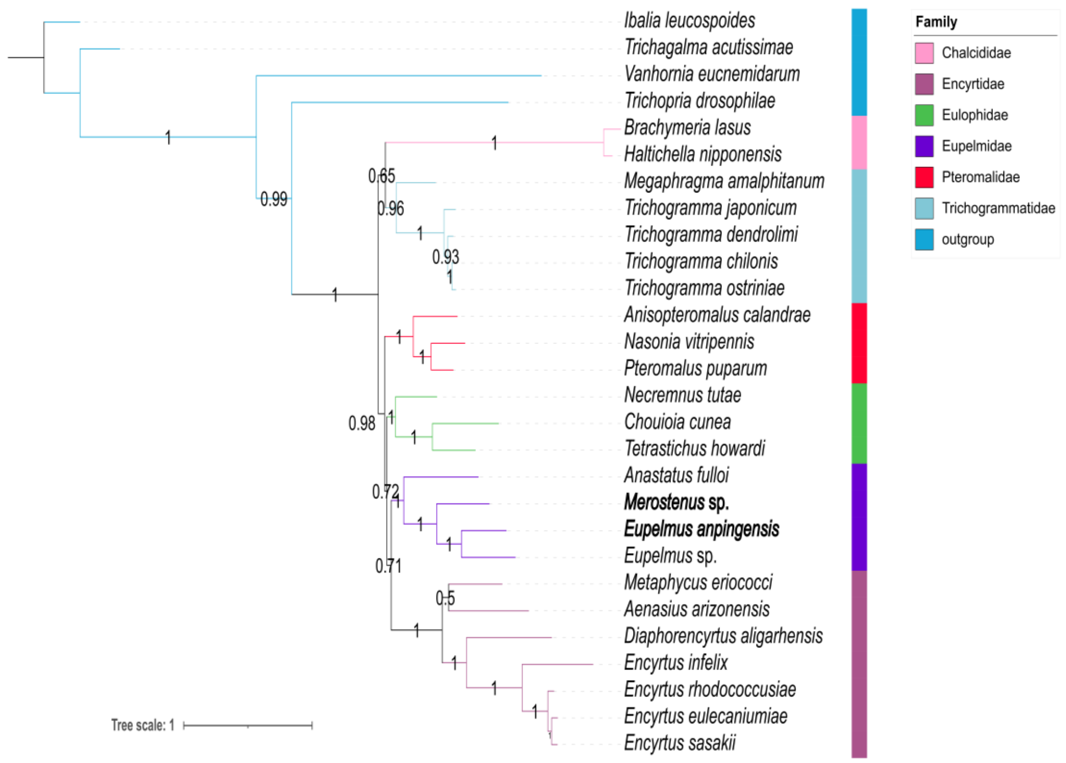

Figure S5. Phylogenetic tree inferred from PhyloBayes based on PCGs dataset. Supports at nodes are Bayesian posterior probabilities (PP).

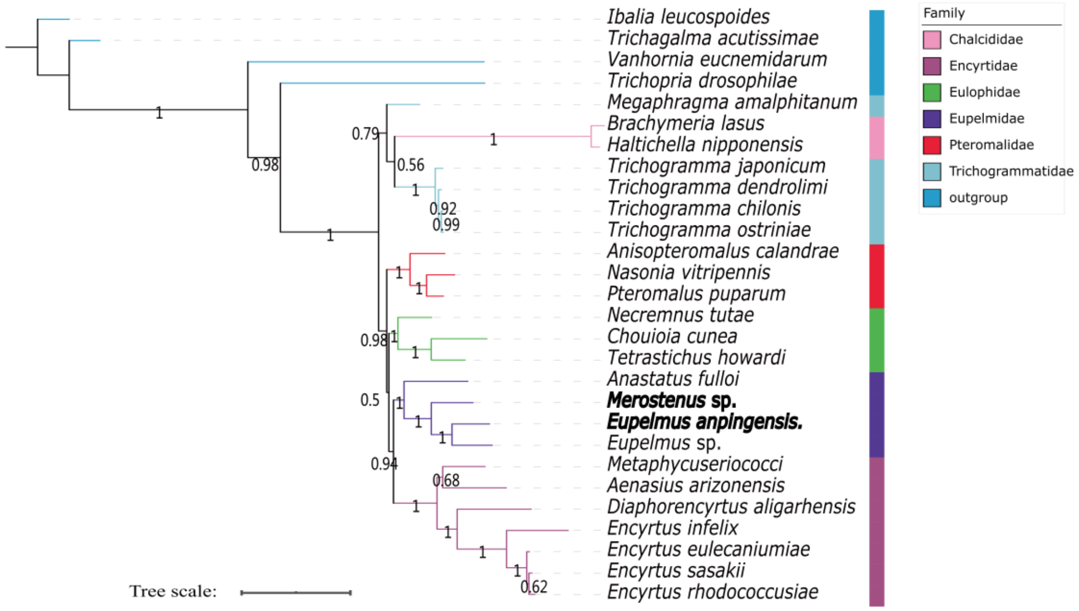

Figure S6. Phylogenetic tree inferred from PhyloBayes based on PCG12 dataset. Supports at nodes are Bayesian posterior probabilities (PP).

Table S1. Partition strategies used in phylogenetic analysis under site-homogeneous models.

Software	Datasets	Partitioning scheme	Models	Software	Datasets	Partitioning scheme	Models
MrBayes	PCGs	P1: cox2; cox3; atp6	GTR+I+G	IQ-tree	PCGs	P1: cox2; cox3; atp6	GTR+I+G
		P2: :nad2; atp8	GTR+I+G			P2: nad2; atp8	GTR+I+G
		P3: cox1	GTR+I+G			P3: cox1	GTR+I+G
		P4: cytb	GTR+I+G			P4: cytb	GTR+I+G
		P5: nad4l; nad4; nad5; nad1; nad3	GTR+I+G			P5: nad4l; nad4; nad5; nad1; nad3	GTR+I+G
		P6: nad6	GTR+I+G			P6: nad6	GTR+I+G
	PCG12	P1: atp6; cox3	GTR+I+G		PCG12	P1: atp6; cox3	GTR+I+G
		P2: atp8; nad6; nad2	GTR+I+G			P2: atp8; nad6; nad2	GTR+I+G
		P3: cox1	GTR+I+G			P3: cox1	GTR+I+G
		P4: cox2	GTR+I+G			P4: cox2	GTR+I+G
		P5: cytb	GTR+I+G			P5: cytb	GTR+I+G
		P6: nad4l; nad1; nad3; nad4; nad5	GTR+I+G			P6: nad4l; nad1; nad3; nad4; nad5	GTR+I+G
	PCG123RNA	P1: cox2; cox3; atp6	GTR+I+G		PCG123RNA	P1: cox2; cox3; atp6	GTR+I+G
		P2: nad2; atp8	GTR+I+G			P2: nad2; atp8	GTR+I+G
		P3: cox1	GTR+I+G			P3: cox1	GTR+I+G
		P4: cytb	GTR+I+G			P4: cytb	GTR+I+G
		P5: nad4l; nad4; nad5; nad1; nad3	GTR+I+G			P5: nad4l; nad4; nad5; nad1; nad3	GTR+I+G
		P6: nad6	GTR+I+G			P6: nad6	GTR+I+G
		P7: rrnL; rrnS	GTR+G			P7: :rrnL; rrnS	GTR+G
	PCG12RNA	P1: atp6; cox3	GTR+I+G		PCG12RNA	P1: atp6; cox3	GTR+I+G
		P2: atp8; nad6; nad2	GTR+I+G			P2: atp8; nad6; nad2	GTR+I+G
		P3: cox1	GTR+I+G			P3: cox1	GTR+I+G
		P4: cox2	GTR+I+G			P4: cox2	GTR+I+G

	P5: cytb	GTR+I+G		P5: cytb	GTR+I+G
	P6: nad4l; nad1; nad3; nad4; nad5	GTR+I+G		P6: nad4l; nad1; nad3; nad4; nad5	GTR+I+G
	P7: rrnL; rrnS	GTR+G		P7: rrnL; rrnS	GTR+G
AA	P1: atp6; nad1; cox3; cox2; cytb; nad3	MTREV+I+G	AA	P1: atp6; nad1; cox3; cox2; cytb; nad3	MTREV+I+G
	P2: nad5; nad4; atp8	MTREV+I+G		P2: nad5; nad4; atp8	MTREV+I+G
	P3: cox1	MTREV+I+G		P3: cox1	MTREV+I+G
	P4: nad2; nad6; nad4l	MTREV+I+G		P4: nad2; nad6; nad4l	MTREV+I+G

Table S2. Features of mitochondrial genome of *Eupelmus anpingensis*.

Name	Strand	Location	Length(bp)	Start codons	Stop codons	Intergenic sequence(bp)
trnI(gat)	J	1-67	67			0
D-loop	J	68-298	231			0
trnM(cat)	J	299-366	68			-1
trnV(tac)	J	366-430	65			138
rrnS	J	569-1212	644			3
trnQ(ttg)	J	1216-1284	69			0
trnA(tgc)	J	1285-1353	69			11
rrnL	J	1365-2656	1292			-21
trnL1(tag)	J	2636-2701	66			6
nad1	J	2708-3646	939	ATA	TAA	2
trnS2(tga)	N	3649-3715	67			71
cob	N	3788-4921	1134	ATG	TAA	1
nad6	N	4923-5423	501	ATT	TAA	108
trnT(tgt)	N	5532-5597	66			12
trnP(tgg)	J	5610-5676	67			-15
nad4L	J	5662-5952	291	ATT	TAA	0
nad4	J	5953-7281	1329	ATG	TAA	-1
trnH(gtg)	J	7344-7405	62			0
nad5	J	7406-9088	1683	ATT	TAA	-1
trnF(gaa)	J	9088-9150	63			39
trnE(ttc)	N	9190-9219	30			11
cox1	J	9231-10763	1533	ATG	TAA	14
trnL2(taa)	J	10778-10842	65			51
cox2	J	10894-11565	672	ATT	TAA	0
trnK(ttt)	N	11566-11630	65			-1
trnD(gtc)	J	11630-11693	64			24
atp8	J	11718-11879	162	ATT	TAA	-1
atp6	J	11879-12553	675	ATG	TAG	0
cox3	J	12554-13339	786	ATG	TAG	233
trnG(tcc)	J	13573-13635	63			2
trnR(tcg)	J	13638-13703	66			30
nad3	J	13734-14078	345	ATT	TAA	-17
trnC(gca)	J	14062-14123	62			26
trnS1(tct)	J	14150-14211	62			19
trnY(gta)	J	14231-14295	65			8
trnN(gtt)	N	14304-14371	68			11
trnW(tca)	N	14383-14447	65			-2
nad2	N	14446-15453	1008	ATT	TAA	26

Table S3. Features of mitochondrial genome of *Merostenus* sp.

Name	Strand	Location	Length(bp)	Start codons	Stop codons	Intergenic sequence(bp)
trnI(gat)	J	1-64	64			0
D-loop	J	65-765	701			1002
trnM(cat)	J	1768-1836	69			6
trnV(tac)	J	1843-1907	65			-23
rrnS	J	1885-2659	775			-6
trnQ(ttg)	N	2654-2720	67			10
trnA(tgc)	J	2731-2796	66			23
rrnL	J	2820-4108	1289			-23
trnL1(tag)	J	4086-4149	64			0
nad1	J	4150-5085	936	ATA	TAA	2
trnS2(tga)	N	5088-5153	66			11
cob	N	5165-6307	1143	ATG	TAA	-1
nad6	N	6307-6810	504	ATT	TAA	42
trnT(tgt)	N	6853-6917	65			25
trnP(tgg)	J	6943-7005	63			-12
nad4L	J	6994-7281	288	ATT	TAA	-1
nad4	J	7281-8615	1335	ATG	TAA	0
trnH(gtg)	J	8616-8679	64			54
nad5	J	8734-10350	1617	ATT	TAA	2
trnF(gaa)	J	10353-10414	62			21
trnE(ttc)	N	10436-10498	63			0
cox1	J	10499-12031	1533	ATG	TAA	2
trnL2(taa)	J	12034-12099	66			0
cox2	J	12100-12786	687	ATT	TAA	-13
trnK(ttt)	N	12774-12838	65			-1
trnD(gtc)	J	12838-12902	65			5
atp8	J	12908-13069	162	ATT	TAA	-1
atp6	J	13069-13743	675	ATG	TAA	-1
cox3	J	13743-14528	786	ATG	TAA	6
trnG(tcc)	J	14535-14597	63			6
trnR(tcg)	J	14604-14668	65			22
nad3	J	14691-15041	351	ATT	TAA	0
trnC(gca)	J	15042-15108	67			-2
trnN(gtt)	N	15107-15171	65			0
trnS1(tct)	J	15172-15228	57			0
trnY(gta)	J	15229-15294	66			-2
trnW(tca)	N	15293-15355	63			-2
nad2	N	15354-16358	1005	ATT	TAA	12
